# Viral-Immune Cell Interactions at the Maternal-Fetal Interface in Human Pregnancy

**DOI:** 10.3389/fimmu.2020.522047

**Published:** 2020-10-07

**Authors:** Elaine L. Parker, Rachel B. Silverstein, Sonam Verma, Indira U. Mysorekar

**Affiliations:** ^1^Department of Obstetrics and Gynecology, Washington University in St. Louis School of Medicine, St. Louis, MO, United States; ^2^Department of Pathology and Immunology, Washington University in St. Louis School of Medicine, St. Louis, MO, United States

**Keywords:** NK cells, T cells, macrophages, Hofbauer cell, human cytomegalovirus, decidua, pregnancy, placenta

## Abstract

The human decidua and placenta form a distinct environment distinguished for its promotion of immunotolerance to infiltrating semiallogeneic trophoblast cells to enable successful pregnancy. The maternal-fetal interface also successfully precludes transmission of most pathogens. This barrier function occurs in conjunction with a diverse influx of decidual immune cells including natural killer cells, macrophages and T cells. However, several viruses, among other microorganisms, manage to escape destruction by the host adaptive and innate immune system, leading to congenital infection and adverse pregnancy outcomes. In this review, we describe mechanisms of pathogenicity of two such viral pathogens, Human cytomegalovirus (HCMV) and Zika virus (ZIKV) at the maternal-fetal interface. Host decidual immune cell responses to these specific pathogens will be considered, along with their interactions with other cell types and the ways in which these immune cells may both facilitate and limit infection at different stages of pregnancy. Neither HCMV nor ZIKV naturally infect commonly used animal models [e.g., mice] which makes it challenging to understand disease pathogenesis. Here, we will highlight new approaches using placenta-on-a-chip and organoids models that are providing functional and physiologically relevant ways to study viral-host interaction at the maternal-fetal interface.

## Introduction

Pregnancy is a unique immunological phenomenon in which the semiallogenic fetus is able to grow in the maternal uterine environment. In order for a successful pregnancy to occur, healthy placentation is necessary to create an environment that is protective for the developing fetus and promotes growth. How immune balance is maintained by maternal and fetal cells to promote the survival of the genetically distinct fetus, while preventing infection by a large number of pathogens, is yet to be fully elucidated (1). This little understood enigma has been the subject of interest and research for decades (2).

**Figure 1:**
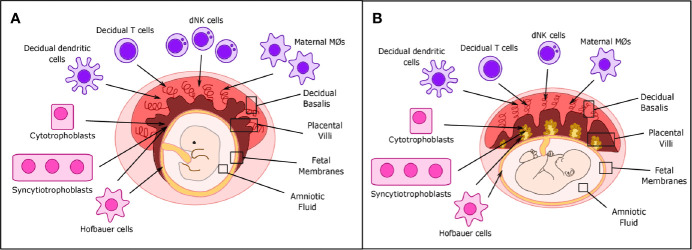
Placental structure and location of decidual immune cells in placental tissues. **(A)** During the first trimester, decidual NK cells, T cells, and dendritic and maternal macrophages are located primarily in the decidua basalis, while fetal Hofbauer cells (HCs) are primarily located in the placental villi and fetal membranes. Cytotrophoblast (CTBs) can be found in both the decidua basalis and placental villi, while syncytiotrophoblasts (STBs) are only found in the placental villi. **(B)** In term placentas, location of decidual immune cells is roughly the same, but the number of maternal macrophages and decidual Natural Killer (dNK) cells can be reduced.

Fertilization leads to the creation of single celled embryo which undergoes several successive divisions to form a blastocyst. The blastocyst is made up of two types of cells: the outer trophoblast or trophoectoderm (TE) layer forming the placenta and chorion, and the inner layer or inner cell mass (ICM) forming the embryo proper and amnion (3). The decidua underlying the embryo is called the decidua basalis, which composes the maternal side of the placenta. The maternal-fetal interface is made up of the maternal decidua and fetally-derived placenta. During implantation, the blastocyst attaches to the decidualized endometrium and the outer layer of the blastocyst differentiates into different lineages. The TE gives rise to cytotrophoblast cells (CTBs) which follow villous and extravillous pathways to form the placenta. In the villous pathway, the mononuclear CTBs fuse, creating multinucleated syncytiotrophoblasts (STBs) that establish floating villi (FV). The FV are surrounded by maternal blood, with STBs aiding the provision of nutrients by enabling gas exchange and exchange of secreted pregnancy-related hormones (human chorionic gonadotropin, hCG, human placental lactogen, hPL) at the maternal-fetal interface. Furthermore, CTBs act as anchoring villi for the attachment of the embryo to the uterus. The CTBs present in the cell column of the anchoring villi follow the extravillous pathway and differentiate into interstitial (iCTBs) and endovascular extravillous trophoblast cells (eCTBs). The iCTBs further invade up to the inner third of the myometrium and eCTBs remodel the spiral arteries in low resistance high blood flow to provide nutrients to the developing embryo (3–6). The invasion of trophoblast cells at the maternal-fetal interface occurs in the presence of a large population of maternal immune cells (7). This includes 70% decidual Natural Killer (dNK) cells, 20%–25% macrophages, 3%–10% T cells and 1.7% dendritic cells (8–10). The abundance of decidual cytotoxic T cells and macrophages can vary through the course of pregnancy (11). The abundance of NK cells in the decidua during the first trimester, and through the pregnancy (albeit at lower abundance), implicates them as an essential element in both the promotion of an immunotolerant environment and the control of pathogenic infection during pregnancy (**Figure 1**). Thus, the paradoxical maternal-fetal interface is admired for both its immunotolerance to semiallogeneic trophoblastic invasion (leading to a successful pregnancy) while remaining remarkably resilient to pathogenic infections. Nevertheless, several pathogen, termed TORCH pathogens (described below), successfully cross the placental barrier and cause devastating infection in the developing fetus (12). In this review, we will look at the interactions between decidual immune cells and specific viral TORCH pathogens and review known mechanisms which may enable viral pathogenesis within the placental environment.

TORCH is an acronym defining some of the most common infections associated with vertical transmission. Initially described in 1971, this group contained just 4 pathogens; Toxoplasmosis, Rubella, Cytomegalovirus (CMV) and Herpes simplex type 1 and 2 (13). Since then this group has been broadened to comprise a host of other infections including *Listeria monocytogenes*, Syphilis, Varicella Zoster virus, Human immunodeficiency virus (HIV), enteroviruses and parvovirus B19 (14). Most recently, following the Zika virus (ZIKV) epidemic in South America resulting in observed congenital anomalies, this group has been further expanded to include ZIKV, with some suggesting renaming this group “TORCHZ” (15). The mechanism by which these “TORCHZ” pathogens are able to circumvent typical clearance by groups of immune cells (e.g. NK cells, macrophages and others) has been studied by many groups over the last few decades in order to elucidate not only routes of pathogenicity but also roles of immune cells within this immune-privileged environment (12). It remains to be proven whether the new emerging viral threat by SARS-COV2 which causes COVID-19 including in pregnant women, will be included in this group of vertically transmitted pathogens (16).

**Figure 2:**
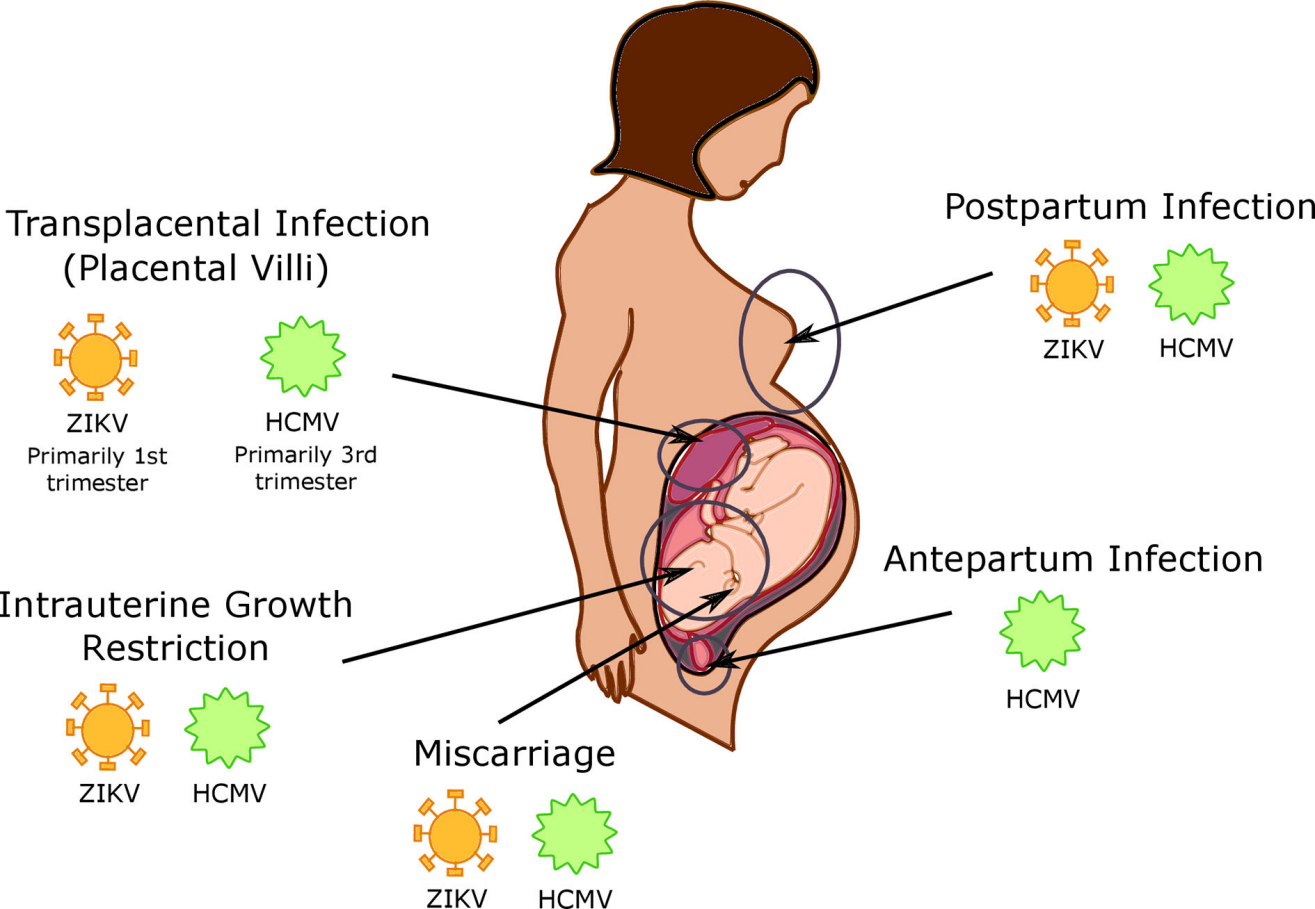
Timing and location of viral infections in the placenta. Human cytomegalovirus (HCMV) is capable of intrapartum infection, as well as postpartum infection during breastfeeding; it is currently unknown whether Zika virus (ZIKV) is capable of this type of transmission as well. HCMV and ZIKV are both capable of transplacental infection, with HCMV infection primarily occurring in the 3^rd^ trimester and ZIKV infection is more common in the 1^st^ trimester. CMV and ZIKV infection additionally cause both intrauterine growth restriction and miscarriage.

In this review, we will focus on maternal and fetal macrophages, T cells, and NK cells and their relationship with each virus. We will focus on the viruses human Cytomegalovirus (HCMV) and ZIKV, which are known causes of adverse pregnancy outcomes and delve into how they interact with various decidual immune cells to promote their survival and replication. We will examine the timings of pregnancy that appear to be most permissive to pathogenic infection by these viruses and we will look at the role of various immune cells in this context (**Figure 2**).

## Decidual T Cells

In the early first term decidua, 3%–10% of resident leukocytes are T cells with approximately 30%–45% of these T cells being CD4+ (T helper cells) and 45%–75% being CD8+ (cytotoxic) T cells (17–19). Further studies have estimated the decidual CD4+ population to be comprised of about 50% activated memory CD25dim T cells and 5% CD4+ CD25bright FOXP3+ Treg cells. Unlike the peripheral circulation, the decidua has a higher ratio of CD8+ T cells to CD4+ T cells and an overall higher number of CD8+ T cells (20). In addition, approximately 40% of the decidual CD8+ population are effector-memory T cells with reduced perforin and granzyme B in comparison to their peripheral counterparts (21). One study published in 2016 described a small percentage of CD8+ T cells found in uncomplicated term decidua to be viral specific. Though these populations of viral specific CD8+ T cells were 1.3% and 2.2% in the decidual basalis and decidual parietalis respectively, they demonstrated that this was higher than that seen in peripheral blood and postulated a role for their presence in the decidua as one of immunoprotection for the fetus. This study could not conclude upon the origin of these T cells, and whether they were recruited from the periphery or activated in the decidua. In addition, more work remains to be done to establish whether these virus specific CD8+ T cells exist early in pregnancy (22). Another study has described the presence of a small population of CD4+ HLA-G+ T cells which are thought to acquire HLA-G through trogocytosis from decidual dendritic cells. It is thought that these T cells promote immunotolerance at the maternal-fetal interface, and they have been shown to be downregulated in pathologies such as preeclampsia (PE) (23). Therefore, it appears T cells play specific roles in immunity and tolerance. To this end we will look at the role that various populations of T cells may play in either enabling or preventing infection by TORCH pathogens at the maternal-fetal interface.

## Macrophages—Maternal and Fetal

Macrophages constitute 20-25% of all leukocytes in the first trimester decidua and play an important role in tissue remodeling, angiogenesis, host defense and immunotolerance (24). Macrophages are considered a key link between adaptive and innate immunity, communicating to other immune cells and modulating their activity (25, 26). These cells are therefore vital throughout pregnancy, adapting their phenotype to address the changing requirements of the evolving decidua (27). Tissue resident decidual macrophages are thought to be recruited from monocytes in the peripheral circulation (28). Distinct subtypes of macrophages have been shown to be present in first-trimester decidual tissue exhibiting immunomodulatory, proinflammatory, and tissue remodeling phenotypes and play key roles in protective immunity as well as fetal tolerance (29). Decidual macrophages are known for their highly immunosuppressive phenotype at the maternal-fetal interface, expressing CD206, DC-SIGN and Tim-3 among other receptor markers (30, 31).

In addition to these maternally derived macrophages exist fetal-derived macrophages called Hofbauer cells (HCs), which sit in the stroma of the chorionic villi (32). These HCs are resident in close proximity to fetal vessels and trophoblast cells from the first trimester until birth. HCs could serve as a portal of entry for pathogens from the infected mother (33). Initially during implantation, they appear to have an inflammatory M1 phenotype which has both microbicidal activity and promotes a cell-mediated Th1 cytokine response. Later, they shift to a mixture of both M1 and M2 phenotypes following trophoblastic invasion and remodeling (34, 35). Several studies have implicated HCs in host viral interactions. Here, we look at the reciprocal interactions between HCs, maternal macrophages, and HCMV and ZIKV.

## NK Cells

The NK cell population in the peripheral circulation is predominately made up of CD56dim CD16+ cells, which are believed to have a more cytotoxic phenotype (36). Approximately 10% of the peripheral circulation is constituted by CD56bright NK cells, which have a more immunotolerant phenotype (37). In the decidua, these NK cell proportions are reversed; 70-80% of the total lymphocytes are CD56bright CD16- (36). Research has demonstrated a number of dNK subsets within the CD56+CD16- population. It is believed that this distinct immunotolerant population is fundamental to the maintenance of a successful pregnancy, with research postulating both an ability to enable the semiallogenic fetus to thrive while at the same time responding to pathogenic infections. These NK cells reside in the decidua basalis close to invading EVTs and express specific receptors (e.g. KIR receptors, CD94/NKG2A, ILT2) to activate or inhibit EVT function (38). This large population of dNK cells are known to be sustained during the first and second trimester, with their numbers declining toward term (11, 39). Despite the unique immunotolerant phenotype demonstrated by dNK cells, it is evident that this cell population displays a high level of plasticity, gaining cytotoxic function in the presence of specific pathogens (39). One way by which this happens is through activation of dNK cell cytotoxcity *via* killer cell Ig-like receptor 2DS1 (KIR2DS1). Reduced expression of this receptor has been associated with adverse pregnancy outcomes such as miscarriages and fetal growth restriction and individuals with increased KIR2DS1 expression have shown better outcomes post-viral infections (40). We will explore further the role that NK cells play in specific viral infections in pregnancy

## TORCH Pathogens

### HCMV

Human cytomegalovirus (HCMV) was first described in 1954 by Margaret Smith, who replicated a virus from two newborn babies who had died from cytomegalic inclusion disease (CID) (41). What we now know as HCMV first came to the attention of Ribbert et al. in 1881, where intranuclear inclusions within large cells were noted in renal and parotid gland cells of stillborn fetuses. These inclusions, often described as ‘owl’s eye inclusions’, were noted to be surrounded by a clear halo (42). HCMV was identified in the 1950s when Smith, Weller and Rowe isolated and cultured HCMV from salivary glands, adenoid tissue and liver biopsies respectively (43, 44). Mechanisms of vertical transmission of HCMV can either be transplacental during gestation or transvaginal during parturition; additionally, there is some evidence for breastmilk transmission (45). HCMV infection is most likely to occur in the third trimester, demonstrating a 30% risk of mother to child transmission in the first trimester compared to a 70% risk in the third trimester (46–48). Congenital HCMV has been estimated to affect 5–20 in every 1,000 live births, with 10% of HCMV positive infants suffering neurological consequences from birth (49). HCMV infection during pregnancy therefore poses a substantial risk to the developing fetus, leading to congenital disease including cerebral abnormalities such as periventricular calcifications, microcephaly, visual impairment, sensorineural hearing loss, neurodevelopmental delay and hepatomegaly (45). Congenital HCMV affects 20,000–40,000 pregnancies annually in the United States and accounts for 25% of all incidents of pediatric sensorineural hearing loss (50–52). It is estimated that the burden of morbidity associated with congenital HCMV infection is greater than that of other common congenital pediatric conditions such as down’s syndrome or fetal alcohol syndrome (53–55). HCMV is also associated with intrauterine growth restriction and miscarriage. There is a great need to understand maternal immunity pathways involved in HCMV infection to develop effective vaccines **(**56**)**.

**Figure 3:**
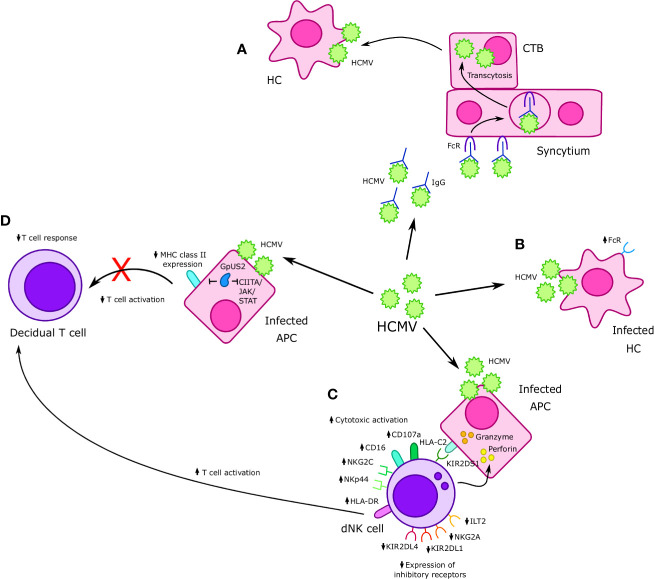
Interactions between Cytomegalovirus (CMV) and immune cells at the maternal-fetal interface. **(A)** Human cytomegalovirus (HCMV)-IgG complexes undergo FcR mediated transcytosis in the syncytium and infect cytotrophoblast (CTBs); HMCV then goes on to infect Hofbauer cells (HCs) in the placental villi. **(B)** HCMV infects HCs, resulting in increased FcR expression. **(C)** Formation of an immunological synapse between decidual Natural Killer (dNK) cells and HCMV infected APCs prompts the release of perforin and granzyme into infected APCs and results in increased expression of cytotoxic receptors NKp44 and NKG2C, as well as other markers of cytotoxicity such as CD16 and CD107a. Furthermore, increased cytotoxicity is accompanied by a reduction in expression of inhibitory receptors such as NKG2A, KIR2DL1, and KIR2DL4. Interaction between KIR2DS1 present on dNK cells and HLA-C on APCs increases dNK cell cytotoxicity in the context of HMCV infection. HMCV infection also results in increased expression of HLA-DR, which may increase T cell activation. **(D)** APCs infected with HCMV exhibit reduced MHC II expression, which is believed to in turn cause a reduced CD8+ T cell response in cases of vertical transmission. The HCMV protein GpUS2 is also believed to downregulate MHC II expression and CIITA/JAK/STAT signaling.

HCMV is associated with asymptomatic infection of most of the world’s population and subclinical illness in pregnant mothers. In the US, an estimated 2% of unexposed pregnant women experience primary infection during pregnancy, resulting in congenital infection in 32% of cases from this population (53, 57–61). However, vertical transmission of HCMV is not only seen in mothers with primary infection but also IgG seropositive mothers, who exhibit a 1% rate of congenital HCMV infection. Mechanisms of infection have been studied through analysis of placental tissue from all three trimesters of human gestation. In placental tissues from those suffering from HCMV, necrosis and oedema has been noted associated with severity of congenital disease symptoms. It has also been noted that HCMV infection is often associated with bacterial coinfection with a potentially pathogenic synergism (62). HCMV resides in the chorionic villi, specifically infecting CTBs, STBs and HCs. It is believed that the ability to travel between STBs in the decidua is key to HCMV pathogenesis (63). Many studies have explored the role of the adaptive and innate immune system in HCMV infection. Below we review established interactions between HCMV and immune cells (**Figure 3**).

### HCMV and Macrophages

HCMV’s ability to infect different populations of macrophages has been demonstrated by several studies. HCMV has been shown to be sequestered by HCs, with placentas from confirmed cases of HCMV infection demonstrating significant hyperplasia of this cell population (64–66). A study investigating vaccine development showed that when neutralizing antibodies are produced against HCMV, rates of HCs infection are decreased (67). A different study utilizing placental explants showed HCMV-IgG immune complexes to undergo Fc Receptor mediated transcytosis as a mechanism to traverse the syncytium to CTBs. HCMV is then taken up by HCs in the placental villi (68). Furthermore, another study by Loenen et al., supports the idea that HCMV genes are able to increase FcR expression on infected cells (69). Another study suggested that HCMV replication in STBs is upregulated in the presence of macrophages (70) by analyzing HCMV replication in STBs alone or when infected STBs were cultured with uninfected placental macrophages. This study also demonstrated elevated levels of HCMV viral titres in co-cultured supernatants when compared to those from STBs cultured alone. This demonstrates that not only do macrophages have the capacity to be infected by HCMV, but also that they may amplify HCMV infection of surrounding cells in the decidua. Some studies have depicted a role for latently infected maternal decidual macrophages in congenital HCMV infection, describing how microbial infections or insults in the placenta may reactivate these macrophages and in turn reactivate HCMV infection (71–73).

### HCMV and T Cells

The maternal-fetal interface is unique in respect to allogenic interactions with CD8+ T cells. EVTs are known to invade the decidua, evading destruction despite the intrinsic ability of CD8+ T cells to recognize foreign antigen *via* MHC class I molecules. As discussed previously, one mechanism by which EVTs are believed to evade CD8+ T cell recognition is through a lack of expression of HLA-A and HLA-B, which are key to CD8+ cytotoxic activity. During pregnancy, many viruses have been shown to upregulate maternal CD8+ T cell activity, leading to migration of highly differentiated effector memory T cells to the decidua. Despite many descriptions regarding the role of T cells in HCMV infection in the fetus and the mother, there are few studies identifying their tissue specific role at the maternal-fetal interface (74).

HCMV is thought to limit CD8+ T cell activity through restriction of MHC class II expression on APCs, which in turn may prevent activation of CD8+ and CD4+ T cells (69). This is thought to be mediated through the HCMV protein GpUS2, which may degrade MHC class II glycoproteins or disrupt downstream CIITA/JAK/STAT signaling pathways (69). Crespo et al., in 2016 demonstrated that HCMV did not induce a significant difference in HLA-G expression on either JEG-3 cells or primary EVTs. HLA-G expression has been associated with immunotolerance, and therefore its persistence despite infection may act to protect infected trophoblast cells from cytotoxic destruction (40).

Studies looking at the role of T cells in viral infection at the maternal-fetal interface demonstrated lower T-cell numbers and response in mothers who vertically transmitted HCMV to their offspring when compared to infected mothers who did not transmit HCMV, potentially suggesting an active role for T cells in vertical HCMV transmission (75).

More specifically, a reduction in the number of CD4+CD45RA+IFN-γ+ Treg cells and CD8+CD45RA+IFN-γ+ T cells in mothers who transmitted HCMV to their fetus was noted when contrasted with mothers who were HCMV positive but did not transmit the infection. There was also a measurable blunted T cell response in HCMV infected mothers who vertically transmitted infection, compared to infected mothers who did not transmit the virus (76, 77). In infected mothers, HCMV virus specific T cells have been shown to be elevated in the final trimester when compared to uninfected mothers (78).

### HCMV and NK Cells

Congenital HCMV infection risk is highest for the fetus in the third trimester, with a 72% transmission risk compared to a 30% risk in first trimester (46). This is despite the abundance of immune cells, specifically NK cells, in early pregnancy. dNK cells exposed to HCMV infected decidual fibroblasts are known to alter their phenotype to express higher levels of activating receptors (such as NKG2D and CD94/NKG2C or NKG2E). Uniquely, utilizing *in vitro* studies, it was noted that decidual NK cells had targeted cytotoxic activity against HCMV infected autologous decidual fibroblasts and heterologous uninfected fibroblast cells, but appeared to spare trophoblast cells (79, 80). This demonstrates a clear cytotoxic effector response by decidual NK cells to HCMV, switching from their typically immunotolerant phenotype with high levels of inhibitory receptor expression (CD94/NKG2A, LIR-1, KIRS), to a cytotoxic phenotype (79, 80). This group also studied the interaction between dNK and HCMV-infected cells using HCMV positive and HCMV negative decidual villous explants. This investigation revealed through fluorescent staining of dNK cells that co-localisation of dNK cells to cells throughout the HCMV positive explant occurred, including synaptic connections which was not seen in HCMV negative explants. This was thought to suggest that the dNK cells were unable to connect with uninfected trophoblasts. This also demonstrates that dNK cells are able to localize and target HCMV infected cells while sparing fetal derived semiallogenic trophoblast cells (80).

dNK cells are unique in their function, both contributing to immunotolerance at the maternal-fetal interface, thereby enabling invasive trophoblastic activity, as well as controlling pathogenic infection (81). This is thought to be mediated by secretion of specific cytokines (79, 82–85). The relatively limited vertical transmission of HCMV during the first trimester of pregnancy, when the population of NK cells is abundant, has led many to speculate about the role NK cells may play in HCMV control (10). Tilburgs and colleagues have recently demonstrated distinct cytotoxic responses in dNK cells to HCMV in first trimester versus at term wherein term pregnancy dNKs harbor reduced efficacy in responding to HCMV–infected cells (86). Siewera et al., suggested that dNK cells undergo a phenotypic transformation to acquire cytotoxic function in the presence of HCMV-infected cells (80). This study proved, through antibody mediated abrogation of the Fas ligand (FasL) and tumor necrosis factor-related apoptosis-induced ligand (TRAIL) on dNK cells, that death of HCMV infected cells is not initiated by dNK cells through these death receptor-ligand pathways. However, this study demonstrated that dNK cells form immunological synapses with HCMV infected fibroblasts, enabling the delivery of perforin/granzyme for cellular destruction. Furthermore, the ability of dNK cells to degranulate in the presence of HCMV infected fibroblasts was demonstrated to be through high levels of CD107a expression, a key cell surface molecule in the mechanism of lytic granule release. dNK cells have also been found to secrete higher quantities of granulysin when compared to peripheral blood NK cells. Upon incubation with infected fibroblast cells, it was noted that CD56bright NK cells decreased from 76.3% to 48%, while there was an elevation in Cd16 expression by NK cells, denoting a transformation to a cytotoxic phenotype. HCMV infected cells have been noted to upregulate expression of natural cytotoxicity receptor (NCRs) NKp44 by almost 2-fold on dNK cells as well as increasing expression of NKG2C. NCRs are associated with activation of the cytotoxic profile of NK cells. Accompanying this was a reduction in NKG2A, KIR2DL1, KIR2DL4, and ILT2 receptor expression, receptors aligned with NK effector inhibitory function (76).

Activating dNK cell receptors such as KIR2DS1, KIR2DS2, KIR2DS5 and KIR3DS1 have been correlated with antiviral activity (40). A study by Crespo et al., demonstrated an increased population of KIR2DS1 + NK cells in the decidua, suggesting an increased activating dNK cell capability in response to HLA-C2, and thereby increased cytotoxic potential. These cells also displayed higher levels of cytolytic molecules when compared to peripheral NK cells. This study demonstrated that KIR2DS1 + dNK cells showed increased cytotoxicity to HCMV infected decidual stromal cells (DSCs) positive for HLA-C2 when compared to KIR2DS1- dNK cells. This was not the case for infected JEG-3 and primary EVT cells, which did not appear to initiate degranulation or cytokine secretion from dNK cells. Despite this, a reduction in the number of infected EVTs in the presence of co-cultured dNK cells was noted, suggesting that dNK may be clearing virus infected EVTs by other means (40). HCMV has been seen to reduce expression of MHC class I, thereby potentially evading CD8+ T cell destruction (87–89). One study reports an initial reduction in HLA-C expression on EVTs in HCMV infection. The possible reason for this is not clear, however this study suggests it could prevent inhibition of NK cells through the HLA-C/KIR2DL1 route, with an additional suggestion of potentially other unknown ligands being upregulated for activation of KIR2DS1, leading to cytotoxic action against infected cells (40).

Another study showed that the potential effect of dNK cell activation on T-cell activation could be mediated *via* an upregulation in HLA-DR expression upon exposure to HCMV infected fibroblasts (80). Therefore, dNK cells may play a role in congenital HCMV infection by potentially protecting the first trimester fetus from infection *via* activation of T cell function.

Collectively, these studies indicate varied interactions between dNK cells and HCMV, with many routes by which HCMV may evade clearance as well as a number of ways through which dNK cells may be activated in the presence of HCMV infected cells. Additionally, dNK cells are seen specifically to modulate activity in the context of T cell activation.

## ZIKV

Zika Virus (ZIKV) was first isolated in 1947 in Zika Forest, Uganda, from infected Rhesus monkey serum during epidemiological yellow fever research (90, 91). However, the first case of human infection was not reported until 1954, when three patients presented with jaundice and were later confirmed to have rising levels of Zika antibodies (92). Initially, ZIKV was associated with innocuous prodromal illness on the African and Indian subcontinents transmitted by the *Aedes aegypti* mosquito, leading to an asymptomatic or self-limiting course of infection (93). In 2007, a mild disseminated infection was identified to be ZIKV in over 70% of the population of the Island of Yap (94). Concerns regarding human ZIKV infections were not aroused until 2013 when incidences of neurological deficits associated with ZIKV infection were first described, with almost 30,000 recorded infections noted in French Polynesia (95, 96). Shortly following this in 2015, a ZIKV epidemic began in South America where not only were neurological deficits such as Guillain-Barré syndrome seen, but also spontaneous abortion and congenital malformations such as microcephaly in infants from infected mothers (91). By the end of the 2017 epidemic in Brazil, there were more than 200,000 notifications of ZIKV cases (97). Estimates for infants born with congenital Zika syndrome (CZS) after the 2015-2016 epidemic ranged from 5 to50 in every 10,000 births (98). The threat of a ZIKV epidemic lingers, with WHO reporting 61 countries affected by *Aedes aegypti* mosquitoes, therefore carrying the potential for ZIKV infection and transmission (99). ZIKV demonstrated continuing global epidemic capacity in India in 2018 (100).

ZIKV belongs to the flavivirus family alongside West Nile virus, Dengue virus, and Yellow fever virus. ZIKV is an enveloped and icosahedral virus with a nonsegmented, 10.7 kb single stranded positive sense RNA genomes (101). This virus is composed of several proteins, categorized as three structural (capsid, pre-membrane and envelope) and seven nonstructural proteins. The seven nonstructural proteins (NS1, NS2A, NS2B, NS3, NS4A, NS4B, and NS5) are essential for viral replication and assembly, as well as being responsible for the pathogenicity of the virus by binding to transcription and restriction factors (95). The biggest risk of congenital ZIKV infection is for mothers infected during their first trimester (102). ZIKV infection demonstrates wide tissue tropism, with ZIKV successfully infecting the central nervous system, blood, retinal, genital and reproductive tissues including placenta (103–107). ZIKV was thought to be exclusively arthropod transmitted until cases of human-human transmission emerged in nonendemic regions, illustrating a role for sexual transmission (108–110). The presence of ZIKV RNA has also been found in breast milk of ZIKV infected mothers (111–113). However, there are reports which suggest that vertical transmission of ZIKV by breastmilk does not occur in most cases, which suggests the possibility that breastmilk does not have a high enough viral load to infect the newborn (114, 115).

**Figure 4:**
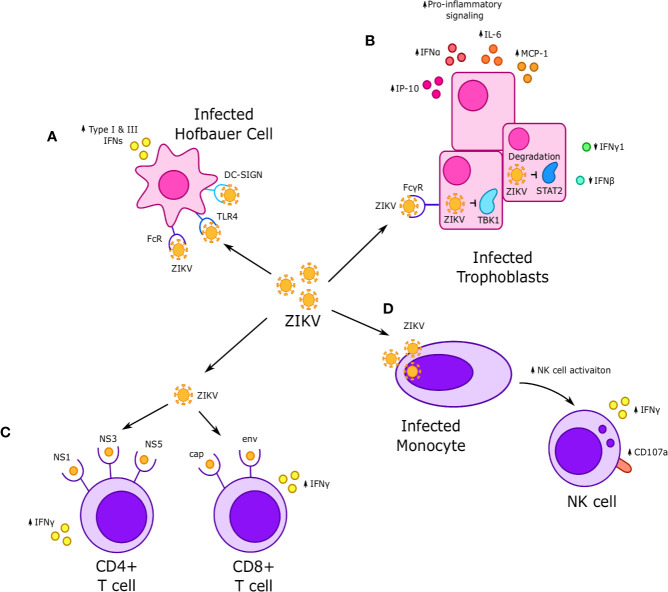
Interactions between Zika virus (ZIKV) and immune cells at the maternal-fetal interface. **(A)** ZIKV infects Hofbauer cells (HCs) and is thought to achieve replication through Fc, TLR4 and DC-SIGN receptors, which in turn increases secretion of type I and III interferons. **(B)** ZIKV infection of fetal trophoblasts and fibroblasts occurs through FcγR and results in increased expression of some interferons, such as IFNα, and decreased expression of others, such as IFN-γ and IFN-β. Infection is associated with increased secretion of proinflammatory cytokines such as IP-10, IL-6, and MCP-1. NS5 viral proteins are thought to downregulate interferon-stimulated genes (ISGs) and reduce interferon signaling *via* STAT2 degradation, while the viral proteins NS1 and NS4B inhibit IFN signaling by downregulating TBK1. **(C)** CD4+ T cells exhibit a strong response to nonstructural NS1, NS3, and NS5 ZIKV proteins, while CD8+ T cells respond to cap and env ZIKV proteins. In both cases, response to ZIKV proteins was characterized by increased IFN-γ production. **(D)** Systemic dNK cells exhibit increased activation, including increased IFNγ production and CD107a expression when incubated with ZIKV infected monocytes.

Despite some knowledge regarding ZIKV pathogenesis, its mechanism of infection in placental immune cell types remains limited (116–119). Histopathology of ZIKV infected placentae has shown ZIKV infection in first trimester villous stromal tissue cells, which includes immune cells in the chorionic villi (117, 120, 121). Uniquely, ZIKV was also found to infect CTBs, endothelial cells, fibroblasts and HC in chorionic villi, as well as amniotic epithelial cells and trophoblast progenitor cells (103, 116–118, 122–124) (**Figure 4**).

### ZIKV and HCs

Similar to HCMV, ZIKV has also been shown to infect HCs and CTBs (90, 116, 117). During the first trimester of pregnancy, ZIKV infects HCs, entering the fetal blood stream in order to reside in the placenta. ZIKV uses HCs as a “Trojan Horse”. This strategy is utilized by several viruses in order to cross the blood brain barrier, where the virus infects leukocytes, leading to them being carried across barriers and thereby enabling the propagation and spread of infection (125–127). HCs have been associated with ZIKV spread to the fetus through the “Trojan Horse” route (91). The presence of ZIKV-specific antigen was demonstrated in HCs in confirmed maternal infection. Multiple studies suggest HCs are a crucial step in vertical transmission of ZIKV to fetal cells, demonstrating that HCs are preferentially infected when compared to CTBs (116, 122, 128). Infection of HCs with ZIKV is thought to propagate infection through hyperplasia and proliferation of these cells, leading to persistence of this HC population into later trimesters (116, 122, 128). A study performed on first trimester fetal and maternal tissue showed that ZIKV can replicate in different cell types, such as decidual fibroblasts and macrophages. It can also infect trophoblasts and HCs as well as umbilical cord mesenchymal stem cells, suggesting that the route of ZIKV infection may move from the decidua basalis to the anchoring villi (129). A study performed using blood from 30+ Asian ZIKV infected pregnant women shows that CD14+ monocytes are the primary target of ZIKV infection. These monocytes are resistant to change in M2 phenotype and downregulate type 1 IFN signaling, which induces the expression of different host genes involved in pregnancy complications (130).

In the *decidua basalis*, ZIKV infects EVTs, macrophages and stromal cells. ZIKV also targets proliferative CTBs in the anchoring villi, however is unlikely to infect STBs due to IFN-λ-mediated antiviral defense mechanisms (131). ZIKV achieves replication within macrophages through FcR, TLR4 and DC-SIGN receptors (116, 132). *In vitro* studies have demonstrated ZIKV infection to be augmented in HCs by IgG from prior flavivirus exposure through antibody dependent enhancement (ADE) (133). There remain many gaps in knowledge regarding the role for macrophages targeted and infected by ZIKV. A study performed using decidual and chorionic villous tissue from early and mid-gestation human pregnancy shows that ZIKV appears to elevate type I and III IFN expression, which does not occur in HCMV infection (131).

### ZIKV and T Cells

Studies looking at the interaction between ZIKV and T cells in humans are scarce although ZIKV infection has been demonstrated to activate both CD4 and CD8 T cells **(**134**)** with specific increases in Vδ2 TCR+ cells which have been implicated in recurrent miscarriages but not associated with ZIKV-induced fetal complications. There have not been notable studies looking specifically at T cell ZIKV communication at the human maternal-fetal interface (135). A recent study examined peripheral T cell responses of 45 confirmed cases of ZIKV infection that had been stimulated with pooled ZIKV peptides from all viral components (136). This study demonstrated responses from both CD4+ and CD8+ T cells to both structural and nonstructural ZIKV components. However, this study particularly showed that CD4+ T cells exhibited a strong response to nonstructural proteins NS1, NS3 and NS5, and CD8+ T cells a strong response to cap and env proteins. This response was demonstrated by marked IFN-γ production from both cell subtypes indicating cell activation (136).

Another case looking at a ZIKV infected individual from the United States demonstrated interactions between the ZIKV NS2 and env proteins with CD4+ and CD8+ T cells, respectively. (137). Furthermore, in a different study, CD4+ T cells of two ZIKV infected individuals showed activity in response to nonstructural proteins (NS1, NS3 and NS5). Consistently, CD8+ T cells were seen to raise activity against the structural protein env (138, 139). These studies demonstrate consistency in the response of CD4+ T cells and CD8+ T cells to ZIKV proteins, revealing CD4+ T cells to specifically respond to particular nonstructural proteins and CD8+ T cells to react to structural proteins, particularly cap and env (136, 138, 139).

Several studies have looked at the ability of DENV-specific CD4+ and CD8+ T cells to be stimulated by the presence of ZIKV peptides in humans (140, 141). These studies showed viral epitopes for specific peptides located in similar regions and structurally conserved across flaviviruses; however, they displayed differences in their sequences (141). Nonetheless, these studies indicated cross-reactivity between the viruses regarding their CD4+ and CD8+ T cell activity. One study demonstrated that CD8+ and CD4+ T cells from DENV positive donors reacted to ZIKV viral peptides, resulting in an upregulation of IFNγ secreting cells. This group also showed that stimulation with ZIKV peptides for those in acute phase of ZIKV infection resulted in recruitment of elevated levels of CD8+ IFN-γ+ T cells (142).

A recent transcriptomics study investigated transcriptional signatures in CD4/CD8 T cells, B, and NK cells and plasmacytoid dendritic cells in patients (nonpregnant) infected with ZIKV **(**143**)**. Interestingly, they did not note significance transcriptional changes in NK or CD8 T cells in a ZIKV infected background but noted significance alterations in pDCs. Whether pregnancy plus ZIKV infection would affect the immune cell transcriptome in humans remains to be determined.

### ZIKV and Peripheral NK Cells

Studies specifically analyzing interactions between dNK cells and ZIKV in humans once again are lacking. However, studies have looked at ZIKV and its communication with peripheral NK cells. One such study postulated crosstalk between monocytes and NK cells in ZIKV infected patients. The activation of NK cells was associated with the presence of monocytes, which induced expression of IFN-γ and CD107a, key markers of NK cell function. Depletion of monocytes in the peripheral blood reduced the levels of these markers and thus the activation of NK cells (144). There are few studies showing the interaction between ZIKV with NK cells. Glasner et al., showed that ZIKV infection led to activation of MHC class I, which was somehow not sensed by dNK cells and their activating receptors, allowing the virus to escape NK cell-mediated killing. MHC class I expression is triggered through the IFN-β pathway *via* activation of RIGI-IRF3 (145). However, the mechanism by which NK cells may promote an immunosuppressive environment in the face of ZIKV infection is not clear. Some studies have indicated that interactions between other aspects of the innate immune system and NK cells may be at play in ZIKV pathogenesis.

### ZIKV and Innate Immunity

There are several studies suggesting that pathogenesis of ZIKV is not mediated through decidual immune cells alone but rather conducted, at least in part, through the activation of interferon-stimulated genes (ISGs), which in turn leads to activation of innate host cell immunity (131). ISGs act to specifically target viral replication. Multiple studies have indicated ZIKV stimulation of interferons (IFN) to vary depending on the type of IFN. While type I and III IFNs have been shown to be inhibited by ZIKV, specifically the NS5 component of the pathogen, Type II IFNs have been shown to be upregulated by the virus (146, 147). One study demonstrated that when Type III IFNs were upregulated, specifically IFN-λ1, trophoblast cells were infected with ZIKV at a lower rate. Further, NS1, NS4A and NS4B have been demonstrated to inhibit IFN type I response. This leads to suppression of the TANK binding kinase 1 (TBK1)/IRF3 and JAK-STAT pathway, which in turn results in reduced activation of innate immune responses (148).

Interferon induced transmembrane protein 1 (IFITM1) and IFITM3 specifically are ISGs which act as restriction factors to inhibit ZIKV replication. The mechanism by which the inhibition and activation of innate immunity impacts the recruitment of innate immune cells to the site of infection is not clear. Little is known about the role of NK cells in human ZIKV infection. One study has noted interactions between TLR7, CD81 and IFITM1, postulating that the restriction of ZIKV is associated with inhibitory activity of IFITM1, potentially through activation of NK cells (149, 150). Another group looking at ISGs showed that viperin played a role in ZIKV pathogenesis, with data revealing that when viperin levels were high, ZIKV mRNA levels were low and vice versa (148). NS4 is seen to target directly the Akt-mTOR pathway, leading to reduced signaling from this pathway and subsequent activation of autophagy in host cells (151). ZIKV has been shown to co-opt the autophagy pathway for post-RNA replication capacity and survival (152, 153). Importantly, the NS2B-NS3 protease activity of ZIKV can be blocked by an inhibitor of autophagy, hydroxychloroquine (HCQ) (154). HCQ is an FDA approved drug considered safe to use during pregnancy and could serve as an effective treatment for preventing ZIKV congenital syndrome (124).

The relationship between ZIKV infected cells and attenuated IFN production has been extensively reported, leading to questions regarding the mechanism underlying this association. It has been proposed that ZIKV may infect cells through ADE of infection. Many cells express the Fcγ receptor, and it is thought that viral particles may complex with antibodies and thereby enter into cells *via* Fcγ receptors (133). Host cells (such as trophoblasts and fibroblasts) infected with ZIKV demonstrate innate immune system activation with a rise in specific IFNs (e.g. IFN-α), but falling levels of others such as IFN-λ1 and IFN-β (155). The elevated levels of proinflammatory cytokines and chemokines, namely IL-6, MCP-1 and IP-10 which are linked to recruitment of immune cells such as monocytes and T cells (155). ZIKV has been shown in multiple studies to downregulate type I IFN signaling and to be active in suppression of antiviral signaling. ZIKV nonstructural proteins NS1 and NS4B inhibit IFN signaling by downregulating levels of TBK1. However, NS2B3 downregulates the JAK-STAT pathway and inhibits apoptosis of ZIKV, and hence inhibits innate antiviral responses (150). One study specifically has implicated the role of the nonstructural ZIKV protein NS5 in promoting ZIKV propagation by targeting STAT2 for degradation, thereby reducing ISG levels (156). This is thought to promote viral replication through a dampened host innate immune cell response. There remains much to be elucidated in terms of ZIKV infection in human pregnancy. New studies are identifying metabolic reprogramming pathways underpinning innate immune responses to ZIKV which opens additional avenues of investigation **(**157**)**. We refer readers to recent reviews highlighting ZIKV-immune interactions in adverse pregnancy outcomes (119) and ADE (158).

### New Tools to Study Viral Interactions at the Maternal-Fetal Interface in Human Pregnancy: Placenta-on-a-Chip and Organoids

The limited availability of placental tissues during early pregnancy has always been a challenge for the reproductive biologists, hampering the study of placental physiology and cell to cell interactions. *In vitro* cell line models can often be biologically distinct and therefore unable to demonstrate enough similarity to replicate the conditions of human pregnancy. In addition, the use of cell line models can fail to reproduce the complexity of the number of cell types and cell interactions present within the decidua. Therefore, functional *in vitro* 3D models being are developed, for example placenta-on-a chip and organoid cultures, which can mimic *in vivo* conditions and would be useful to understand the mechanisms of viral host interactions.

The ‘Placenta-on-a-chip’ is a microfluidics model utilizing human trophoblast cells (BeWo) and fetal derived cells (HUVECs and HPVECs) (159, 160). These cell lines are cultured and separated by a semipermeable membrane within flow conditions with the purpose of understanding placental mechanisms and barrier function (159). Recent reports have described the faithfulness of placenta-on-a-chip model to *in vivo* placental conditions (161). For example, glucose transport using a placenta-on-a-chip model was demonstrated by Lee et al., and Blundell et al., highlighting significant similarity to *in vivo* glucose transport in the human placenta (159, 160). Placenta-on-a-chip models have also been used to investigate the transport of heparin and anti-hyperglycemic agents such as glyburide using BeWo and human placental villous endothelial cells (162). Recently, the transport of the xenobiotic compound caffeine across the placenta has been studied using this model system, providing new insights into the extent of caffeine transfer from mother to fetus (163).

Bacterial infections have also been studied using this model. Zhu et al., showed that in the presence of *Escherichia coli (E. coli)*, trophoblast cells (BeWo) activated the circulating macrophages on the “maternal” side of the chip to secrete several inflammatory cytokines that mimicked *in vivo* conditions during pregnancy (164). The impact of common environmental exposures such as titanium dioxide nanoparticles (TiO_2_-NPs) has also been studied using this 3D placental model showing a series of different placental responses (barrier permeability, oxidative stress, cell apoptosis, and maternal immune cells behavior (165). They showed placental barrier permeability and maternal immune cells to be influenced by even low concentration of NPs (165). Therefore, this simple *in vitro* model can prove useful in understanding the environmental exposure of NPs during pregnancy and can help in a range of biological studies (165). Recent studies report generation of an organ-on-a-chip model, wherein decidualized human endometrial stromal cells and macrophage cell lines are co-cultured in a microfluidic device and shown to inhibit secretion of TNF-α in response to LPS stimulation (166). These devices have also been used to determine the impact of cytokine secretion by dNK cells on the migration of primary trophoblast cells. These studies illustrate the functionality of microfluidic organ on chip devices to elucidate importance of maternal immune cells in the placenta (167). Thus, the use of fetal membrane on organ-on-a-chip provides a suitable model to explore the impact of pathogenic infections during pregnancy (168, 169).

The use of *in vitro* trophoblast organoids as a 3D culture model also provides a new tool to understand the mechanism of implantation at the maternal-fetal interface. Recent studies have shown the characterization of these organoids derived from 1^st^-trimester CTBs (6 to 8 weeks) and suggest their resemblance to primary trophoblast cells (170–172). Due to similarity with the placental architecture, these organoids could be used to study physiological, metabolic and hormonal changes that occur during pregnancy.

The viruses we highlighted in this review, HCMV and ZIKV, do not naturally infect commonly used animal models [e.g., mice] which makes it challenging to understand disease pathogenesis. In particular, there remains a paucity of understanding ZIKV-immune cell interactions during pregnancy. Thus, the employment of placenta-on-a-chip or organ-on-a-chip, and organoid models will be pivotal in providing functional and physiologically relevant ways to study the interaction of immune cells at the maternal-fetal interface with viral pathogens that affect pregnancy.

## Summary

Both HCMV and ZIKV can be sequestered into fetal macrophages. HCMV implicates HCs in the potential infection of other decidual cells, leading to the promotion of HCMV transcytosis in trophoblasts. ZIKV preferentially infects HCs, persisting in this cell population and potentially mediating infection of other fetal-derived cells. More poignant is the suggestion that decidual macrophages may mediate reactivation of HCMV by acting as a latent reservoir for infection. These studies collectively indicate a central role for macrophages in the pathogenesis of TORCH viruses.

dNK cells have been seen to alter their phenotype to express higher levels of various activating receptors when in the presence of infected decidual fibroblast cells. They also are known for their plasticity in the face of specific pathogens, acquiring more cytotoxic function. KIR2DL1/HLA-C2 has been identified as a mechanism by which dNK cells are activated and display cytoxicity toward HCMV infected cells. It has also been suggested that dNK cell activation may trigger activation of T-cells through upregulating HLA-DR expression on infected fibroblast cells.

ZIKV viral components demonstrate capacity to elicit strong responses from peripheral CD4+ and CD8+ T cells, with NS1, NS3, and NS5 being associated with CD4+ stimulation whereas Cap and Env proteins being associated with CD8+. We also see in this review the importance of Interferon stimulating genes in the restriction of ZIKV replication.

**Figure 5:**
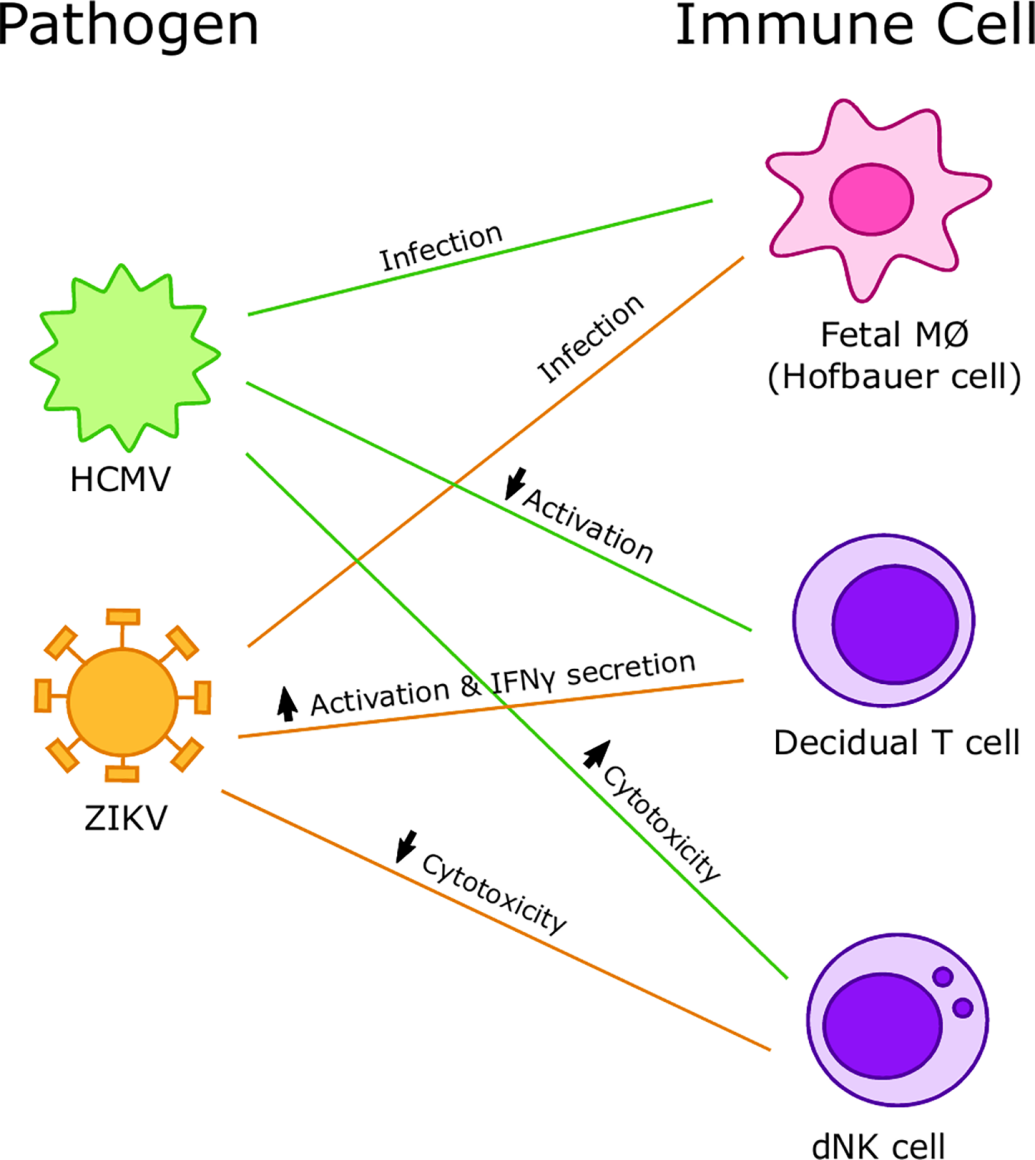
Summary of interactions between viruses and immune cells at the maternal-fetal interface. Human cytomegalovirus (HCMV) preferentially infects Hofbauer cells (HCs) and increases cytotoxicity of decidual Natural Killer (dNK) cells but decreases activation of decidual T cells. Zika virus (ZIKV) preferentially infects HCs and decreases cytotoxicity of dNK cells and increases activation and IFNγ secretion in decidual T cells.

Thus, the implications and outcomes of viral interactions with immune cells at the maternal-fetal interface are varied. We see the importance of the host immune response and recognize the importance of studying mechanisms of pathogenesis in detail to enable targeted therapeutic interventions including vaccines to mitigate the adverse outcomes of viral infections during pregnancy (**Figure 5**). Finally, we posit that better understanding of the immunological underpinnings of infections at the maternal fetal interface can support the inclusion of pregnant women in trials testing vaccines and therapeutics to compact existing and emerging viral infections.

## Author Contributions

EP, SV, and IM wrote the manuscript. RS generated the figures. All authors contributed to the article and approved the submitted version.

## Funding

This work was funded in part by a grant from the National Institutes for Health/National Institute for Child Health and Development R01HD091218 to IM. RS was supported by a MARC uSTAR fellowship.

## Conflict of Interest

IM serves on the Scientific Advisory Board of Luca Biologics.

The remaining authors declare that the research was conducted in the absence of any commercial or financial relationships that could be construed as a potential conflict of interest.
